# Metabolic lesion-deficit mapping of human cognition

**DOI:** 10.1093/brain/awaa032

**Published:** 2020-03-23

**Authors:** Ashwani Jha, Rute Teotonio, April-Louise Smith, Jamshed Bomanji, John Dickson, Beate Diehl, John S Duncan, Parashkev Nachev

**Affiliations:** a1 UCL Queen Square Institute of Neurology, London, UK; a2 Neurology Department of Centro Hospitalar de Leiria, Leiria, Portugal; a3 Institute of Nuclear Medicine, UCL, London, UK

**Keywords:** lesion-deficit mapping, intelligence, depression, ^18^F-FDG PET imaging, epilepsy

## Abstract

In theory the most powerful technique for functional localization in cognitive neuroscience, lesion-deficit mapping is in practice distorted by unmodelled network disconnections and strong ‘parasitic’ dependencies between collaterally damaged ischaemic areas. High-dimensional multivariate modelling can overcome these defects, but only at the cost of commonly impracticable data scales. Here we develop lesion-deficit mapping with metabolic lesions—discrete areas of hypometabolism typically seen on interictal ^18^F-fluorodeoxyglucose PET imaging in patients with focal epilepsy—that inherently capture disconnection effects, and whose structural dependence patterns are sufficiently benign to allow the derivation of robust functional anatomical maps with modest data. In this cross-sectional study of 159 patients with widely distributed focal cortical impairments, we derive lesion-deficit maps of a broad range of psychological subdomains underlying affect and cognition. We demonstrate the potential clinical utility of the approach in guiding therapeutic resection for focal epilepsy or other neurosurgical indications by applying high-dimensional modelling to predict out-of-sample verbal IQ and depression from cortical metabolism alone.

## Teaser

Establishing the functional necessity of a neural substrate requires the study of discrete — usually structural — lesions. Jha *et al.* present a new approach, employing localised metabolic lesions associated with focal epilepsy to derive novel functional anatomical maps of cognition and affect in the human brain.

**See Forkel and de Schotten (doi:10.1093/brain/awaa060) for a scientific commentary on this article**.

## Introduction

Our knowledge of the macroscopic organization of brain function is largely built on functional imaging, now conveniently summarized in meta-analytic databases (Laird *et al.*, 2005; Robinson *et al.*, 2009; Yarkoni *et al.*, 2011; Gorgolewski *et al.*, 2015). For all their number and consistency, such correlative studies cannot distinguish brain regions necessary for a given behaviour from those whose activity is merely coincident with it (Rorden and Karnath, 2004). Furthermore, where a global multifaceted ability—such as intelligence—is indexed by distributed phasic, task-related neural activity, both positive and negative correlations with performance are equally interpretable: the former as ‘enhanced recruitment’, the latter as ‘enhanced efficiency’ (Nachev *et al.*, 2009). These inferential vulnerabilities not only undermine claims about brain function, they also hinder attempts to derive clinically predictive models.

The strongest evidence for the necessity of a brain region for a given behaviour is the emergence of a specific deficit following its inactivation. In humans, such ‘lesion-deficit’ mapping tends to rely on the most common form of focal brain injury: stroke-induced ischaemic damage (Bates *et al.*, 2003; Karnath *et al.*, 2004; Glascher *et al.*, 2010; Mah *et al.*, 2014; Xu *et al.*, 2017). Though ischaemic lesions are abundant, the complex spatial covariance structure imposed by their vascular origins has recently been shown to distort lesion-deficit maps derived with conventional inferential techniques.

Within the mass-univariate frameworks in widespread use, critical brain regions are, at best, statistically indistinguishable from areas of collateral damage within the same vascular territory, and at worst, falsely attributed to non-critical substrate (Inoue *et al.*, 2014; Mah *et al.*, 2014; Xu *et al.*, 2017).

Even when a spatial locus is accurately identified, the critical neural substrate may be remote from it, inactivated through white matter disconnection rather than direct injury. The authority of current stroke-based lesion-deficit maps has consequently been eroded, ceding power to high-dimensional multivariate models capable of neutralizing the distorting effects of the lesion architecture, and of identifying multifocal, network-based patterns of neural dependence (Yourganov *et al.*, 2015, 2016; Pustina *et al.*, 2018). The success of such models, however, is highly sensitive to the scale of available data, which often sets a hard upper limit on real-world applicability.

The problem high-dimensional modelling mitigates is best avoided altogether. Here, we introduce a novel alternative approach to lesion-deficit mapping that amplifies its inferential power while reducing the risk of spatial covariance-induced distortion.

Instead of structural lesions, we use metabolic lesions—focal areas of chronic cerebral hypometabolism, identified with interictal ^18^F-fluorodeoxyglucose PET (^18^F-FDG PET), that often occur in patients with non-lesional focal epilepsy and are reasonably presumed to reflect underlying cerebral focal hypofunction (Rosenow and Lüders, 2001).

Unlike structural lesions, metabolic lesions are defined downstream of the pathological process that produces them, integrating specifically at the cortical level both direct and connective causes of neural dysfunction. It is reasonable to expect the spatial dependencies here to follow the structure of the underlying functional anatomy, for each focal metabolic defect is rendered visible through dysfunction of the functionally-connected neural network it impairs as a whole. As cerebral glucose metabolism is a continuous variable, lesions can be meaningfully modelled as graded deviations from expected activity, a refinement of the crude binary approach structural lesions compel. Not only hypofunction but also abnormal or compensatory hyperfunction may be revealed. In common with all clinical populations, the distribution of dysfunction will not be homogeneous, and interference from non-anatomically distributed pathological factors such as level of education cannot be fully eliminated, but appropriate modelling can mitigate such nuisance effects.

Here we develop this approach within a population with focal epilepsy under the care of our unit, and apply it to the mapping of multiple subdomains of affect and cognition.

A cohort of 159 individuals was identified in whom no structural brain lesions were detectable on high-resolution MRI. Neuropsychological evaluation and ^18^F-FDG PET are routinely performed in these circumstances in consideration of possible neurosurgical treatment of the epilepsy.

We sought to quantify the relation between focal cortical metabolism and neuropsychological scores within two distinct sets of voxel-wise ‘metabolic lesion-deficit’ models, generating inferential maps of the neural dependents of distinct cognitive and affective subdomains, and high-dimensional predictive maps of individual cognitive performance. Our objective is to derive a robust new anatomical map of human cognition, validated—and rendered potentially clinically useful—by its individually predictive power.

## Materials and methods

### Cohort

We retrospectively reviewed imaging and clinical data on 189 patients undergoing clinical evaluation for epilepsy surgery at the National Hospital for Neurology and Neurosurgery, Queen Square, between June 2006 and February 2011. The decision to operate is informed by a comprehensive standardized set of imaging, clinical and neuropsychological assessments, allowing us to survey a relatively unbiased sample. We identified a cohort of patients with no evidence of structural pathology on high-resolution 3 T MRI, in whom interictal ^18^F-FDG PET imaging had been performed, and contemporaneous neuropsychological data were available. Thirty individuals were excluded because the ^18^F-FDG PET was either technically inaccessible or of poor quality. The remaining 159 cases underwent subsequent analysis.

The median age at the time of cognitive testing was 32.0 [standard deviation (SD) = 9.5] years. Forty-nine per cent (78/159) of patients were male, 111 patients (70%) were right-handed, 16 (10%) were left-handed, three (2%) were ambidextrous and the handedness of the remaining 29 was missing from the psychological record. This work has received ethical approval from the HRA and the local research ethics committee for consentless analysis under irrevocable anonymization. This study is reported in accordance with the STROBE checklist (von Elm *et al.*, 2007).

### Neuropsychology

The neuropsychological evaluations were undertaken on clinical grounds as part of presurgical investigations. A common set of neuropsychological instruments were flexibly deployed as dictated by the identified cognitive deficits. Raw test scores were tabulated from the handwritten instrument forms. Data were analysed with custom scripts in MATLAB 2015b (The MathWorks Inc, Natick, MA, USA). Of the 47 different instruments used in these subjects, we selected 16 commonly used measures that also provide a reasonably wide psychological coverage. These tested four broad psychological areas: components of the Wechsler Adult Intelligence Scale III (WAIS) (Wechlser, 2008), memory (list learning, design learning, Warrington Recognition Memory test for Words, Warrington Recognition Memory test for Faces) (Warrington, 1984), fluency (phonemic and semantic), and affect (using the Hospital Anxiety and Depression Scale (HADS) (Zigmond and Snaith, 1983) (Supplementary Table 1). A mean of 76.3% (SD 14.1) of patients underwent each psychological test. Therefore, we developed a robust strategy for dealing with missing data so as to minimize bias. A complete-case approach to missing data—where only subjects with full datasets are analysed—is both inefficient and prone to producing biased results (van der Heijden *et al.*, 2006). We singly imputed missing psychological data using probabilistic principal component analysis (PPCA) (Ilin and Raiko, 2010). PPCA is a standard multivariate technique that extends traditional PCA by adding a Gaussian noise term (Tipping and Bishop, 1999). This allows the principal components to be calculated in the presence of missing data by iterative maximization of the log-likelihood of the PPCA model. Once the PPCA model is estimated, missing data can be back-projected from the components. We chose PPCA, rather than other missing data approaches, because it is more accurate in highly correlated datasets (such as psychological scores) and more robust to a non-random distribution of missing data (Dray and Josse, 2014).

### Imaging

#### Acquisition

^18^F-FDG PET/CT data were acquired on GE Discovery ST and GE Discovery VCT PET/CT scanners. Thirty minutes after the injection of 250 MBq ^18^F-FDG, a CT for attenuation correction was acquired followed by 15 min of PET acquisition. The resulting PET data were reconstructed using ordered-subset expectation maximization (OSEM) iterative reconstruction (three iterations, 20 subsets) with CT attenuation correction and subsequently filtered with a 4 mm Hanning filter to form trans-axial PET images with a 1.95 × 1.95 × 3.27 mm voxel size.

#### Imaging data processing

All imaging data were analysed using SPM12 (http://www.fil.ion.ucl.ac.uk/spm/). The source ^18^F-FDG PET acquisitions were converted to NifTI (Neuroimaging Informatics Technology Initiative) format and non-linearly registered to a standardized ^18^F-FDG PET template that has been validated in the presence of brain atrophy in a dementia cohort (Della Rosa *et al.*, 2014; Perani *et al.*, 2014). Registration was performed using SPM12’s ‘old normalise’ algorithm, which performs a 12-parameter affine registration followed by a separate estimation of non-linear deformations defined by a linear combination of 3D discrete cosine transform basis functions (Ashburner and Friston, 1999, 2000). This algorithm was chosen over the ‘new normalise’ algorithm, which relies on prior grey and white matter maps optimal for images where grey/white contrast is maximal (for example T_1_ MRI) rather than ^18^F-FDG PET. Images resliced to 3 mm isotropic voxels were written in ICBM/MNI space, their intensities adjusted for the degree of non-linear spatial warping (i.e. modulated for volume changes).

Prior to statistical analysis, images were masked to include only consistently high-intensity voxels falling within grey matter. We used a binary optimal-threshold mask designed to maximize the correlation between the included voxels and the average ^18^F-FDG PET image: this has been shown to reduce false negatives in the context of regional brain atrophy (Ridgway *et al.*, 2009).

To account for variations in ^18^F-FDG administered activity, timing of injection, and pharmacodynamics, we estimated the background ^18^F-FDG PET signal per scan, for later use in optimizing the inferential models. For maximum robustness, the background signal was estimated with the aid of measures from high-intensity areas, white matter, and ventricular compartments. High-intensity activity was indexed as the sum of all raw ^18^F-FDG PET counts within the optimal-threshold mask; white matter and ventricular activity as the sum of the voxels falling within each respective tissue compartment (derived from the Neuromorphometrics atlas included with SPM). These three signal estimates—global, ventricular and white matter—were used in subsequent statistical modelling to remove background effects. All images were smoothed with a 6-mm full-width at half-maximum (FWHM) Gaussian filter immediately prior to statistical analysis.

### Statistical analysis

Because the spatial covariance structure of ischaemic lesions has been shown to distort mass-univariate lesion-deficit inference, we first examined whether the spatial covariance structure of the ^18^F-FDG PET signal-derived metabolic lesion maps in this cohort were similarly distorted. Following this, we performed mass-univariate metabolic lesion-deficit inference for a range of neuropsychological deficits, and metabolic lesion-deficit clinical prediction: the former within the SPM statistical framework, the latter with Bayesian multiple regression with Markov Chain Monte Carlo estimation.

#### Spatial covariance modelling

##### Generation of metabolic and ischaemic lesion maps

To isolate the functionally relevant signal, confounding effects were first removed from the raw ^18^FDG PET count data by entering the images into a voxel-wise multiple regression model in SPM with the previous confounding variables alone. The residuals were written as images composed of 3 mm isotropic voxels and, for computational reasons, subsequently smoothed by a 10 mm FWHM Gaussian filter and then downsampled to 10 mm isotropic. The resultant images were masked with the optimal-threshold mask, leaving 1906 grey matter voxels per image. Unlike stroke where voxels are assigned to a binary category (lesioned versus unaffected), metabolic lesions are described on a continuous scale by the degree of metabolism (lesions corresponding to hypometabolism). To allow comparison of the spatial covariance of metabolic lesions with stroke lesions, we generated binary metabolic lesion maps by dichotomizing voxel intensities at a threshold of 2 SD below the median of each voxel (Supplementary Fig. 1) similarly to thresholding procedures in previous clinical studies (Foster *et al.*, 2007). Ischaemic lesion maps were used as a comparator: 1333 automatically segmented, binary stroke lesion maps reported in Xu *et al.* (2017). These images, written as 1 mm isotropic voxels, were identically smoothed with a 10 mm FWHM Gaussian filter, and then downsampled to 10 mm isotropic voxel size. The optimal-threshold mask was thereafter applied so that the resulting voxels were spatially identical with those in the ^18^FDG PET signal-derived metabolic lesion maps

##### Local dependency

**Figure 1:**
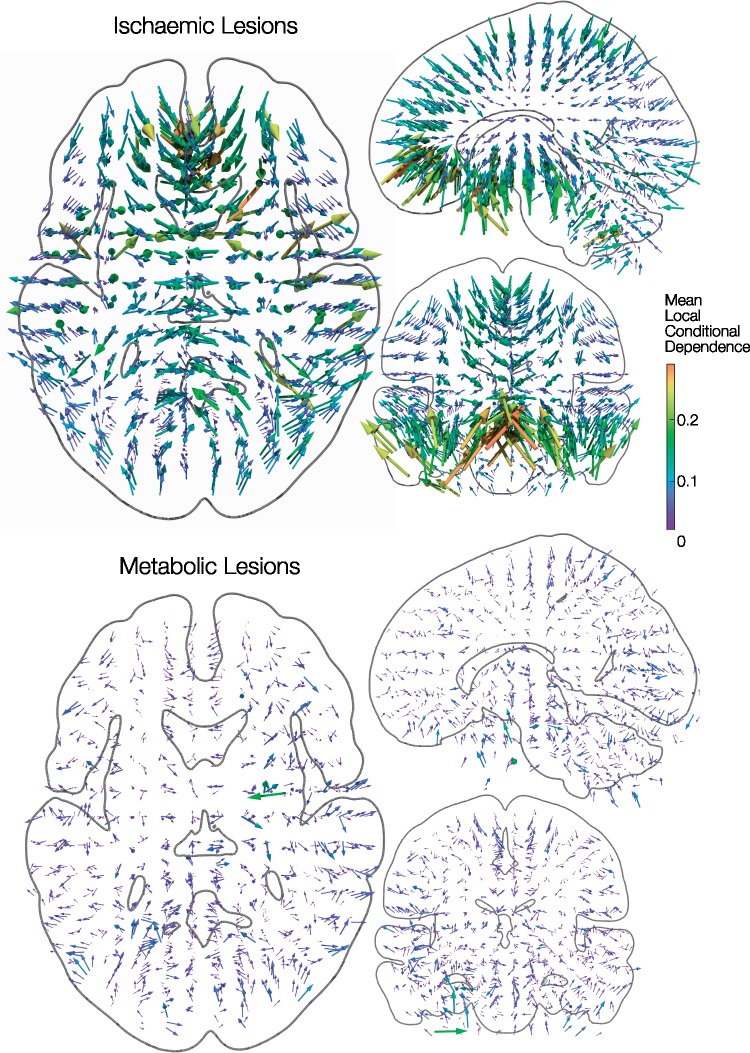
**Covariance structure of metabolic lesions: local dependency.** The short-range lesion-dependency structure is shown for 1333 binary ischaemic (*top*) and 159 binary metabolic (*bottom*) lesion maps. Given any lesioned voxel location, the conditional probability of each of six neighbouring voxels also being affected was summed into a single vector pointing towards the direction of greatest local dependence (and therefore potential inferential distortion). The resultant voxel-wise conditional dependency vectors are 3-dimensionally rendered as arrow glyphs against orthogonal slices through a canonical white matter surface in MNI space. Larger magnitude is represented with warmer colours and larger glyphs. Ischaemic lesions show a striking pattern: lesioned voxels are strongly and systematically influenced by damage to other voxels within the proximal arterial distribution. In contrast, voxels within metabolic lesions show minimal and relatively unstructured local dependencies from which more robust lesion-deficit inferences can be drawn.

Given a lesion involving a specific voxel, the conditional probability of each of six neighbouring voxels also being affected can be empirically calculated and represented as six vectors where magnitude corresponds to the conditional probability, and direction corresponds to the relative location of the neighbouring voxel. The mean of these vectors—the normalized vector sum, also known as the mean resultant length—is a single conditional dependency vector that points towards the expected direction of greatest local dependence (and therefore of potential inferential distortion). The magnitude of this vector is a probability and is, therefore, bounded between 0 and 1. Isotropic local dependencies will result in small randomly distributed mean vectors, whilst anisotropic dependencies will result in larger systematically directed vectors. Voxel-wise conditional dependency vectors were estimated for both metabolic and ischaemic lesions and were visualized to identify local dependency patterns across the brain (Fig. 1), before their magnitudes were subjected to a Kolmogorov–Smirnov test as a proxy for testing the degree of isotropy of different lesion types (Supplementary Fig. 2). Data visualization was performed in Paraview (https://www.paraview.org/).

##### Global dependency

**Figure 2:**
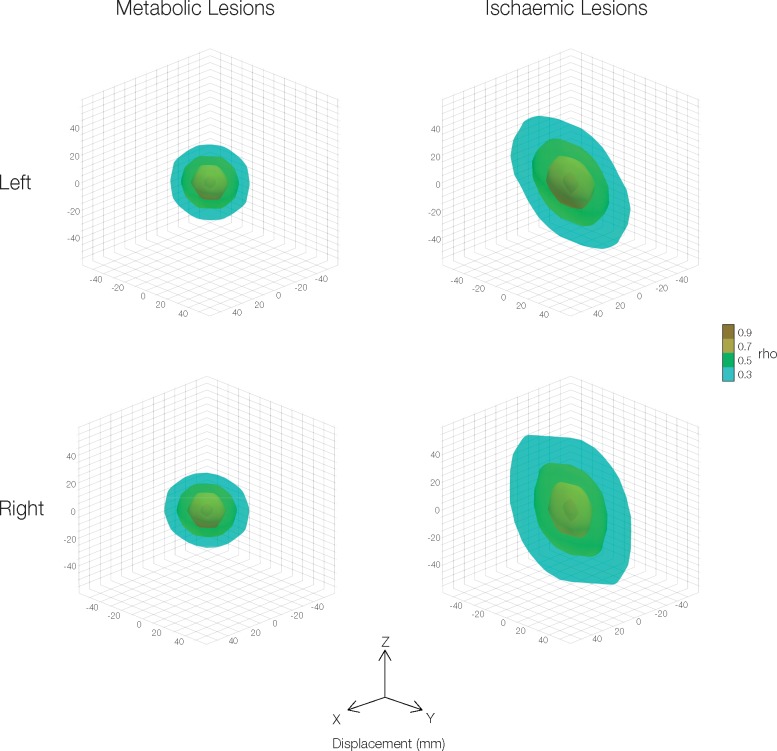
**Covariance structure of metabolic lesions: global dependency.** The long-range correlation structure is shown for 1333 binary ischaemic and 159 binary metabolic lesion maps. To avoid missing potential differential hemispheric biases, the left and right hemispheric voxels are presented separately. The correlation between every pair of grey matter voxels was binned according to degree of displacement in the *x*, *y* and *z* planes. Isocontours of the median correlation coefficient (*rho*) are presented as a function of displacement. Metabolic lesions are spatially isotropic, whilst ischaemic lesions show asymmetric elongated spatial correlations in the *y* and *z* planes especially. It is the anisotropy of the latter that distorts mass-univariate lesions-deficit analysis.

In addition to anisotropic dependencies with directly neighbouring voxels, longer-range voxel-voxel associations may also be anisotropic, again potentially distorting lesion-deficit inference. Given the high number of voxel-voxel comparisons involved, we characterized these associations in a simpler manner by binning each possible pairing of grey matter voxels by their relative Euclidian displacement in three dimensions and then calculating the median voxel-voxel correlation within each spatial bin. The resulting 3D image represents the association (correlation coefficient, *rho*) of any two voxels being affected by the same lesion as a function of their displacement in three dimensions. We postulated that, for metabolic lesions, the sample voxel-voxel correlation would vary uniformly with extended distance over the three spatial dimensions, whereas ischaemic lesions would show a heterogeneous variation across the three planes. Additionally, for this analysis, we separately used voxels from the left and right hemispheres, to avoid missing potential differential hemispheric biases which are often seen in focal pathologies such as ischaemic stroke (Fig. 2). Data visualization was performed in Paraview (https://www.paraview.org/).

#### Mass-univariate functional lesion-deficit analysis

Standard voxel-based morphometry methods were used for the mass-univariate analysis using the original ^18^F-FDG PET images (not the arbitrarily thresholded metabolic lesion maps generated for the investigation of spatial covariance). At each voxel, the ^18^F-FDG PET count, a dependent variable, was entered into a multiple regression with a single variable of interest (a psychological score) and confounding variables as independent variables. Confounding variables were standardized across all models, and included age, handedness, and the three estimates of scan-specific background activity (global, white-matter, and ventricular). After model estimation, two one-tailed *t*-tests were performed on the regression coefficients (slopes), with the resulting SPMs thresholded at *P* <* *0.025 FWE (cluster-based family-wise correction, *P* <* *0.0001 uncorrected cluster forming threshold). The usual threshold alpha (0.05) was halved because two unidirectional tests were performed for each behavioural variable of interest. For simplicity, in the main text we therefore describe this as *P *<* *0.05 FWE two-tailed. The analysis was repeated for each behavioural variable, resulting in a pair of SPMs for each behavioural variable of interest. Anatomical labels were largely based on the peak voxel within the cluster as labelled by the Neuromorphometrics atlas available within SPM12 (http://www.neuromorphometrics.com/), but were manually checked and rationalized by two experienced neurologists. Data visualization was performed in Surf Ice (https://www.nitrc.org/projects/surfice/).

#### Multivariate functional lesion-deficit analysis

We used multivariate analyses to quantify the prediction of psychological scores from ^18^F-FDG PET imaging and to identify the correspondence between mass-univariate and multivariate maps of the same data. A cross-validated Bayesian penalized regression framework was used (BayesReg) (Makalic and Schmidt, 2016). For two example behavioural variables (WAIS verbal IQ and HADS depression score), we individually specified a regression model with the behavioural variable of interest as a dependent variable and the ^18^F-FDG PET data voxels as 1906 independent variables. The large number of independent variables relative to the number of cases impairs the performance of standard regression models, inducing instability and poor generalization. We therefore used penalized regression, which suppresses large model parameter estimates. In the Bayesian setting, this can be done by applying shrinkage priors: a hyperparameter on the regression coefficients whose prior distribution has a substantial mass around zero. We used the default Lasso prior in the BayesReg package, which uses an adaptive Laplace prior distribution over the betas. As high-dimensional models are analytically intractable, marginal likelihoods and posterior parameter estimates were estimated using Markov Chain Monte Carlo (MCMC) sampling using a Gibbs procedure. Posterior parameter values were derived by integrating 5000 MCMC samples [after 1000 samples burn-in and every fifth sample was included (thinning)]. Chains were assessed for adequate convergence. The generalizability of the model predictions was assessed using out-of-sample cross-validation. This was performed by training the model on a randomly selected 85% of the data and assessing predictive performance on the remaining 15%, i.e. a 15% hold-out procedure, which was repeated 50 times each using different random splits of the data. Model accuracy performance is presented as the average out-of-sample root mean squared error over the 50 runs.

### Data availability

Open-source software is available from the resources as cited, or from the authors on request. Summary SPM images are also available but not raw images owing to restrictions arising from patient confidentiality.

## Results

### Spatial distribution of metabolic lesions

Signal-normalized and transformed into standard stereotactic space, the PET images disclosed anatomically circumscribed regions of reduced metabolism naturally intelligible as metabolic lesions. Other than an expected slight predilection for temporal regions, where epileptogenicity is more common, the observed distribution was reasonably uniform in comparison with previously published stroke data (Supplementary Fig. 1). Coverage extended across the entire brain, enabling the evaluation of lesion-deficit relationships at all loci.

### Covariance structure of metabolic lesions

Damage to a given voxel depends on damage to its neighbours: lesions naturally occur in patches. Whereas the local dependencies of focal blood oxygenation level-dependant (BOLD) activation approximate Gaussian fields, structural—especially vascular—lesions are non-randomly shaped by the pathological process. Unless explicitly modelled within a high-dimensional multivariate framework, such non-random dependencies will tend to distort the inferred lesion-deficit relation, in a manner that will depend on the underlying neural substrate of interest and so cannot be trivially corrected (Mah *et al.*, 2014).

Lesioned voxel dependencies may be empirically characterized at two spatial scales: local and global. At the local scale, we may calculate the directional dependence between a given voxel and its six immediate neighbours, across all lesions, yielding a summed vector that points in the direction of greatest dependence and therefore of greatest potential distortion. Where the dependence exhibits no directional preference, the magnitude of this vector will tend to zero, and its direction will be random across voxels; where a directional preference is present, the magnitude will be greater than zero as a function of the extent of dependence and its directionality, and the direction will tend to vary non-randomly across voxels. A comparison of our metabolic lesions with stroke lesions shows substantially less local isotropy of dependence, and therefore less potential distortion (Fig. 1 and Supplementary Fig. 2).

At the global scale, we may calculate the dependence between all pairings of voxels as a function of their proximity, separately for each spatial plane. As in the local case, if the dependence exhibits no spatial bias, its relationship with proximity should be identical across all planes; if not, then the relationship will be exaggerated in the favoured plane. A comparison between the two kinds of lesions again shows substantially lesser, here global, isotropy (Fig. 2).

The maximal permissible anisotropy cannot be formally determined because it depends on the unknown structure of the underlying neural organization. But given that the anisotropy of stroke lesions—the most common aetiology—has been shown to distort even simple hypothetical lesion-deficit relationships, substantially reduced anisotropy ought to enhance the fidelity of the resultant maps.

### Metabolic lesion-deficit anatomical mapping

A comparatively benign lesion covariance structure having been established, we proceeded to derive lesion-deficit maps based on voxel-wise mass-univariate inference. The sample distributions of a broad range of neuropsychological scores yielded sufficient variance to investigate their neural substrates (Supplementary Table 1). Lower cognitive scores generally correspond to greater cognitive impairment except for the HADS scores, where higher scores reflect greater anxiety and depression.

The inferred maps exhibited two cardinal characteristics. First, compared with maps derived from structural lesions (Karnath *et al.*, 2004; Gläscher *et al.*, 2009, 2010) the distribution of critical regions was larger and more widely disseminated. Second, not only positive but also extensive negative correlations of metabolic activity with neuropsychological performance were observed.

#### Wechsler Adult Intelligence Scale

**Figure 3:**
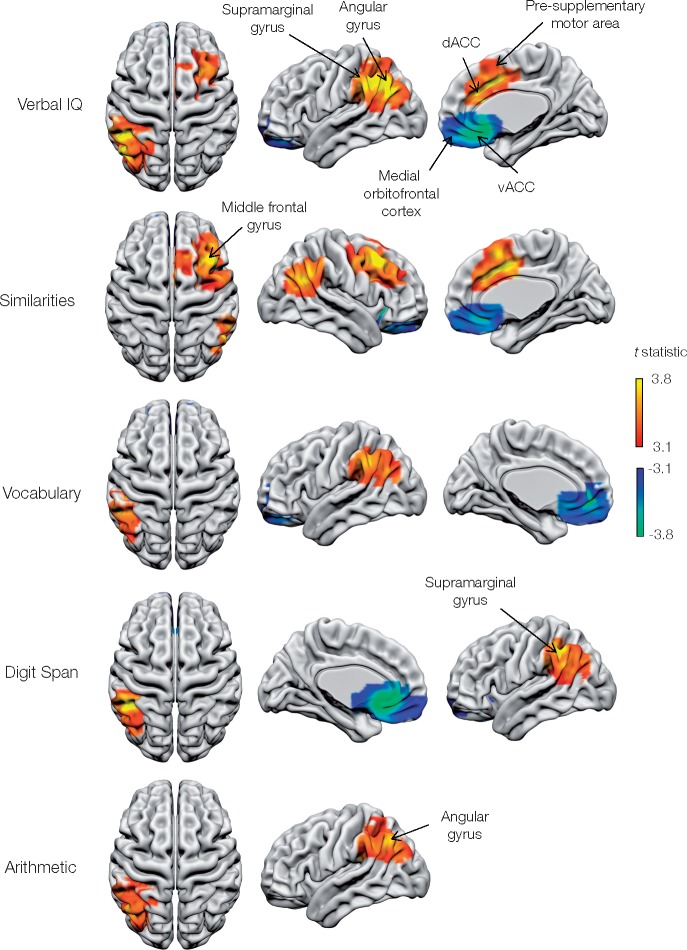
**Metabolic lesion-deficit mapping of the components of the Wechsler Adult Intelligence Scale (WAIS): verbal IQ.** Voxel-wise statistical parametric lesion-deficit maps of WAIS verbal IQ and subcomponents are three dimensionally rendered onto a canonical white matter surface in MNI space. Only voxels surviving the *P *<* *0.05 two-tailed FWE correction for multiple comparisons are shown. Voxels are coloured according to their corresponding *t*-statistic, with positive associations (where hypometabolism corresponds to an impairment of cognitive scores) displayed on a red-yellow scale and negative associations displayed on a blue-green scale. Three different rotations of each map are shown per row of images next to the test labels. dACC = dorsal anterior cingulate cortex; vACC = ventral anterior cingulate cortex.

**Figure 4:**
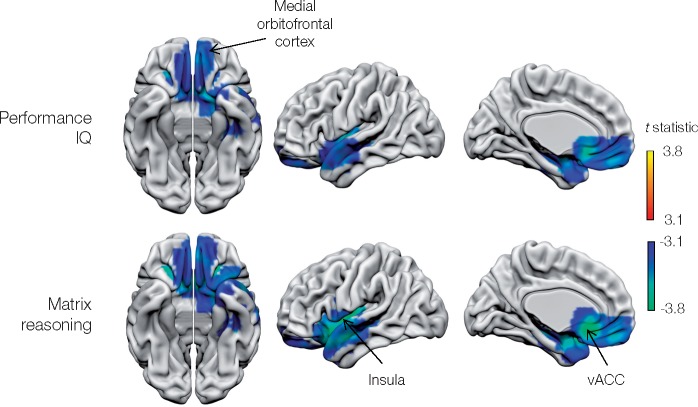
**Metabolic lesion-deficit mapping of the subcomponents of the Wechsler Adult Intelligence Scale (WAIS): performance IQ.** Voxel-wise statistical parametric lesion-deficit maps of the WAIS performance IQ and matrix reasoning subcomponent are shown. Image characteristics and abbreviations are as in Fig. 3.

A core network of strongly lateralized fronto-parietal areas emerged (Figs 3, 4 and Table 1). Critical areas for verbal IQ included the left supramarginal/angular gyrus complex, together with the right presupplementary motor area and middle frontal gyrus. The WAIS-Similarities subtest showed similar neural dependents, but covering a wider expanse of right middle frontal gyrus, and including right angular gyrus rather than the left supramarginal/angular gyrus complex.

**Table 1:** Statistical peak activations from mass univariate analyses

Test group	Test component	Peak voxel, *x y z*	Region	*t*-statistic	*P*
Memory	Design Learning	45 18 33	Right middle frontal gyrus	4.53	<0.001
		48 −51 39	Right angular gyrus	4.32	0.002
		−33 −30 −27	Left fusiform gyrus	−4.65	0.001
	Warrington Recognition Memory test for Words	−39 −42 −12	Left fusiform gyrus / left superior temporal gyrus	4.54	0.004
		−51 −63 −30	Left inferior temporal gyrus	4.50	0.009
		69 −33 18	Right superior temporal gyrus/ middle temporal gyrus	4.24	0.004
	Warrington Recognition Memory test for Faces	60 −27 24	Right supramarginal gyrus, right middle temporal gyrus	4.73	0.001
WAIS	Vocabulary	−48 −48 36	Left supramarginal gyrus	4.32	0.008
		18 27 −9	medial orbitofrontal cortex	−5.45	<0.001
	Similarities	15 15 36	Right pre-supplementary motor area / dorsal anterior cingulate cortex	4.89	<0.001
		51 −60 27	Right angular gyrus	4.26	0.019
		18 27 −9	Medial orbitofrontal cortex	−4.36	0.001
	Arithmetic	−48 −45 33	Left supramarginal gyrus	4.29	0.007
	Digit Span	−48 −45 39	Left supramarginal gyrus / left angular gyrus	4.87	0.002
		6 21 −12	Medial orbitofrontal cortex, ventral anterior cingulate cortex	−5.12	<0.001
	Matrix Reasoning	−39 −3 0	Medial orbitofrontal cortex, left anterior insula	−4.93	<0.001
		−9 27 −9	Ventral anterior cingulate cortex	−4.47	<0.001
	Verbal IQ	−48 −48 36	Left supramarginal gyrus	5.08	<0.001
		15 15 39	Right pre-supplementary motor area / dorsal anterior cingulate cortex	4.78	0.008
		15 27 −9	Medial orbitofrontal cortex / ventral anterior cingulate cortex	−5.01	<0.001
	Performance IQ	−9 24 −9	Medial orbitofrontal cortex, left anterior insula	−4.16	<0.001
		−39 −3 0	Ventral anterior cingulate cortex	−4.03	0.005
HADS	Depression^a^	−30 12 −12	Left anterior insula, ventral anterior cingulate cortex	−5.13	0.001
		−45 −63 9	Left middle temporal gyrus	4.97	0.023
Fluency	Semantic	−45 −84 24	Left middle occipital gyrus	4.18	0.003
		3 18 −12	Medial orbitofrontal cortex	−5.88	<0.001
	Phonemic	−48 −45 39	Left supramarginal gyrus / angular gyrus	4.59	0.001
		54 −36 36	Right supramarginal gyrus	4.36	0.006
		−45 −81 27	Left middle occipital gyrus	4.31	0.000
		3 15 −15	Medial orbitofrontal cortex	−5.53	<0.001

Summary results are presented for each cognitive test. The peak voxel in each cluster that survives *P *<* *0.05 two-tailed FWE correction is shown. The direction of the association is given by the sign of the *t*-statistic. Cognitive impairment is indexed with decreasing cognitive score for all tests. A positive *t*-statistic implies that hypometabolism corresponds to an impairment of cognition.

a Depression is indexed by the HADS in which higher scores are usually pathological, but the score has been reversed to align the interpretation with the other cognitive scores: a positive *t*-statistic implies that hypometabolism corresponds to an impairment of affect.

Attentional aspects of working memory, as indexed by the numerical WAIS-Arithmetic and WAIS-Digit span tests, were limited to the left supramarginal/angular gyrus, more posteriorly than the verbal IQ network.

The activity of a remarkably consistent set of brain regions was negatively related to psychological scores: bilateral medial orbitofrontal cortex for verbal IQ, performance IQ, WAIS-Similarities, WAIS-Vocabulary, WAIS-Matrix reasoning, extending to include left anterior insula and anterior cingulate for performance IQ and matrix reasoning.

#### Memory

**Figure 5:**
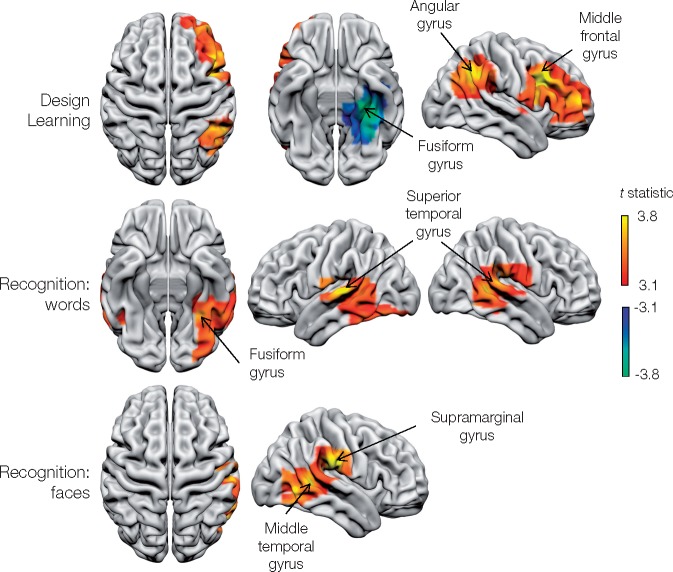
**Metabolic lesion-deficit mapping of memory.** Voxel-wise statistical parametric lesion-deficit maps of individual memory tests. Image characteristics and abbreviations are as in Fig. 3.

A set of lateralized, fronto-parieto-temporal areas was identified as critical to memory as indexed by Warrington Recognition Memory test (Fig. 5 and Table 1). Delayed design recall identified a large frontal area centred on the right middle-frontal gyrus and the right angular gyrus. A negative relationship was found with the left mesial temporal region, centred on the left fusiform gyrus. No significant regional changes in activity were detected for immediate list or design recall, and delayed list recall.

Areas critical to verbal memory were localized to the temporo-parietal junction bilaterally, centred on the superior and middle temporal gyri, and also the left inferior temporal and fusiform gyri. Recognition memory for faces was strongly lateralized to the right temporo-parietal area, involving the right supra-marginal, and middle and inferior temporal gyri.

#### Fluency

**Figure 6:**
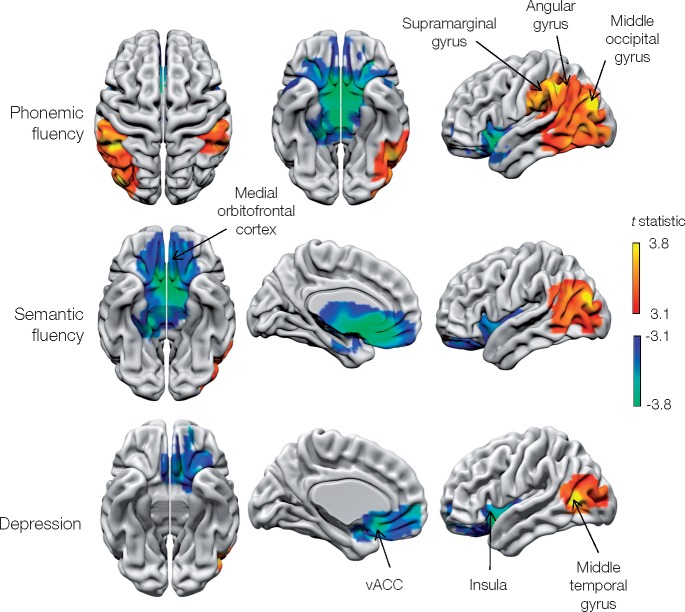
**Metabolic lesion-deficit mapping of fluency and affect.** Voxel-wise statistical parametric lesion-deficit maps of fluency and depression (HADS). Higher depression scores are pathological, but the score has been reversed in the above images to match other psychological scores used: a positive correlation implies that hypometabolism corresponds to an impairment of affect (greater depression) and is shown on a red-yellow scale. Image characteristics and abbreviations are as in Fig. 3.

Phonemic fluency was dependent on activity in bilateral supramarginal gyrus and right angular gyrus (Fig. 6 and Table 1). The only area positively associated with both phonemic and semantic fluency was the left middle occipital gyrus. Both types of fluency, however, were negatively associated with activity in the medial orbitofrontal cortex.

#### Affect

Depression was positively associated with metabolic activity in the left anterior insula and anterior cingulate, and negatively associated with activity in the left middle temporal gyrus (Fig. 6 and Table 1). At least 28 (17.6%) patients were diagnosed as having active, ongoing affective disturbances where treatments including medication or therapy were already being taken or were suggested following psychiatric assessment (Supplementary Table 2). No significant regional changes in activity were detected for anxiety.

### Metabolic lesion-deficit clinical prediction

The cognitive function of any individual patient is plausibly determined by the sum and interactions of a multiplicity of neural substrates. To predict it requires a high-dimensional multivariate approach, modelling the metabolic activity of each voxel in the brain as an independent predictor (Inoue *et al.*, 2014; Mah *et al.*, 2014). The complex multivariate distribution of stroke damage renders analogous models difficult to estimate with structural lesion data (Mah *et al.*, 2014; Xu *et al.*, 2017). We hypothesized that multivariate functional-lesion modelling would be relatively efficient here, requiring significantly fewer images. Focusing on two areas owing to the high computational demands of the approach, we demonstrated the feasibility of predicting WAIS verbal IQ and HADS depression score with high-dimensional models. High-dimensional Bayesian penalized multiple regression of metabolic data predicted individualized out-of-sample verbal IQ and HADS depression score remarkably well, exhibiting out-of-sample root mean squared errors of ±13.8 (SD 1.7) and ±3.2 (SD 0.47), respectively.

**Figure 7:**
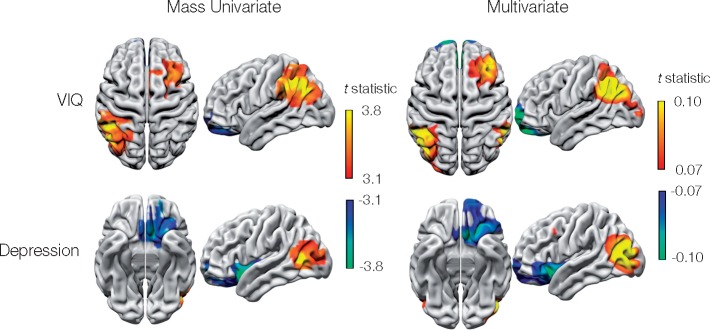
**Metabolic lesion-deficit clinical prediction of WAIS-verbal IQ and depression.** Penalized Bayesian multiple regression was used to predict WAIS verbal IQ (VIQ) and depression (HADS) reasonably well from ^18^F-FDG PET data. Although the model is evaluated in terms of predictive accuracy, it is interesting to compare the support for these multivariate predictions—the weighting of each voxel in the model—with the weightings assigned in the univariate case. Multivariate weightings (*right*) presented as the *t*-statistic are broadly similar to the univariate weightings (*left*, reproduced from Figs 3 and 5), providing further support that the univariate maps represent genuine, undistorted structure-function maps.

The predictive fidelity of the multivariate models lends credibility to their anatomical feature weightings, which closely correspond to those identified in the lesion-deficit anatomical mapping (Fig. 7). A close correspondence is congruent with the benign covariance structure of metabolic lesions observed in the preceding analysis.

## Discussion

Resting metabolic lesion-deficit mapping opens a new perspective on the macroscopic organization of the brain that combines the inferential strength of structural lesion mapping with the spatial resolution and robustness of functional imaging. In common with the former, it identifies regions critical to cognitive function. In common with the latter, it permits simplifying assumptions about the spatial structure of neural patterns to be introduced with less danger of anatomical distortion.

The resultant maps can sustain strong claims to functional necessity naturally confined to grey matter, greatly simplifying the interpretation of disruption that with structural lesions confusingly spans both processing and connective neural substrate.

Applied to the anatomical basis of the cognitive domains, the approach casts further light on an organization increasingly recognized to be more distributed than discrete (Jung and Haier, 2007; Duncan *et al.*, 2000). Recent large-scale structural lesion-deficit analyses have yielded remarkably extensive, spatially continuous patterns, with right insular and temporoparietal cortex critical to perceptual organization, and large areas of left insular and parietal lobe critical to verbal comprehension (Gläscher *et al.*, 2009, 2010). Wernicke’s area in verbal comprehension, and the left angular gyrus in the numerical components of the WAIS, were notably absent in these studies.

One source of the historical diversity of inferred anatomical patterns is the distorting effect of the vascular tree in the dominant lesion aetiology—stroke (Inoue *et al.*, 2014; Mah *et al.*, 2014). The multi-focal network identified in our study may reflect greater robustness to such distorting effects. We found the left inferior parietal lobule (IPL) to be implicated in most verbally tested components of the WAIS, and the only area identified when testing vocabulary only. Components requiring greater manipulation or evaluation of words—verbal IQ and similarities subtests—showed dependence on areas of dorsomedial and dorsolateral frontal cortices. These findings are in accord with the wider neuroscientific literature, which implicates the IPL in auditory and written verbal understanding and semantic interpretation (Stoeckel *et al.*, 2009; Seghier, 2013), the presupplementary motor area in task conditional complexity (Nachev *et al.*, 2008), and the dorsolateral frontal cortex in planning and working memory (Rowe *et al.*, 2000).

The neural dependents of WAIS subsets included bilateral parietal areas, but were strikingly lateralized frontally. That more posterior regions of the IPL, centred on the angular gyrus, were critical to numerically based tests of the attentional aspects of working memory endorsing the long-held relation between this area and numeracy (Seghier, 2013). Fluency exhibited a broadly similar pattern, involving bilateral IPL but also more posterior occipito-temporal regions (Fig. 6).

Within the broad domain of memory, dependence on the right angular gyrus in design learning coheres with lesion studies of spatial attention and neglect (Singh-Curry and Husain, 2009); this task further invoked right prefrontal cortex as one might expect from a load on working memory (Rowe *et al.*, 2000). Bilateral temporo-parieto-occipital areas were implicated in visual recognition of words, and a similar area on the right in face recognition. The left fusiform gyrus was key for word recognition, but was concurrently hypermetabolic when testing in patients with impaired design learning (Fig. 5).

Whereas structural lesions merely identify areas of spatially discrete dysfunction, whole brain ^18^F-FDG PET allows us to identify areas of hyperfunction, proportional to the behavioural deficit. Such activity may be compensatory to a deficit elsewhere, focal preservation in the face of globally reduced function, a pathological correlated reorganization of resting state activity, or subclinical epileptic activity at the time of the scan. As patients were not performing a task at the time of imaging, this phenomenon is not explained by failure of task-related deactivation of the default mode network (Raichle *et al.*, 2001). The medial orbitofrontal cortex, anterior cingulate and left insula were consistently hypermetabolic in subjects with impairment of verbal IQ, performance IQ, vocabulary and matrix reasoning, and phonemic and semantic fluency more or less indifferently to the task. This may be explained by focal preservation of orbitofrontal metabolic signal in patients with hypometabolism elsewhere, which appears as increased signal due to global signal normalization. But given that depression scores were also positively correlated with broadly the same areas, pathological reorganization seems the likeliest explanation.

The predominance of elevated metabolism in depression in our study (Fig. 6) may explain the inconsistency of previously reported structural lesion-deficit localizations (Drevets, 2001; Nickel and Thomalla, 2017). Our findings are of particular interest given that chronic stimulation of the subgenual cingulate is an emerging treatment for depression (Delaloye and Holtzheimer, 2014).

Loss-of-function studies in humans are inevitably complicated by their pathological origins. Though epilepsy may arise from most areas of cortex and is remarkably diverse in its aetiology, even a large, unselected cohort such as ours cannot be expected to sample the full breadth of anatomical substrate evenly, though it is bound to be broader than neurodegenerative, disease-specific studies (Foster *et al.*, 2007; Perani *et al.*, 2014). In spite of this, the distribution of functional lesions in this cohort was reasonably homogeneous (Supplementary Fig. 1). Comparison with binary ischaemic lesions is also complicated by the continuous nature of metabolic lesions, characterized in degrees of dysfunction. This aspect nonetheless improves the statistical efficiency of mass-univariate analysis, and reflects the natural continuity of cognitive function itself.

Patients with epilepsy experience phasic neurological dysfunction—seizures—of variable extent and duration. Focal patterns of cerebral dysfunction could be modified by these, by interictal epileptic discharges, and other global effects such as anti-epileptic medication (Theodore, 1988). We explicitly included linear global confounds in the statistical models to account for such effects. The observed neuropsychological patterns across the cohort exhibit similar structure to the wider population, suggesting that the fundamental neural mechanisms are likely to be broadly representative. Equally, task-related functional imaging studies in epilepsy patients do not support any major reorganization of the neural substrate, certainly not at the scale of interest here. Finally, large clinically acquired datasets inevitably suffer from heterogeneously collected and missing data, here manifest by individual clinical selection of appropriate cognitive tasks for each subject. These concerns were mitigated as scores were recorded by a small cadre of clinically highly-trained neuropsychologists using standardized cognitive tasks designed for patients considering epilepsy surgery. Furthermore, we used a robust approach to non-random missing data—imputation with probabilistic PCA—to ensure that our findings were protected from bias as far as possible.

While the focus here has been on improving the principal current concern of lesion-deficit inference—its specificity—future work should aim to improve its sensitivity. No critical regions were detected for some psychological elements, such as anxiety scores, and compared with ischaemic lesion maps some regions were notably absent (such as Wernicke’s and Broca’s area for the verbal IQ). This may be due to non-linear relationships between metabolism and psychological score, the opportunity for neural adaptation to chronic slowly evolving metabolic lesions, or lack of psychological variation within our cohort. Alternatively, the broad cognitive and affective behavioural measures used may poorly represent neural architecture in isolation: verbal IQ is mediated by educational attainment (and vice versa) and verbal IQ or its subtests are not designed to measure verbal skills well in isolation of IQ. Because most of these elements are highly correlated, they cannot be entered together as independent variables for lesion-deficit mapping and so require an intermediate data compression step such as factor analysis or ideally an informed generative model of the psychological attribute in question.

## Conclusion

We have developed a novel metabolic lesion-deficit mapping technique based on interictal ^18^F-FDG PET in patients with focal epilepsy. Unlike conventional structural lesion-deficit mapping, our approach is not distorted by vascular territories, yielding arguably the first spatially unbiased estimates of brain regions critical to a broad range of psychological subdomains underlying affect and cognition. Not only do these maps provide powerful evidence of the underlying functional specialization of the human brain, they also provide the potential to be developed into a clinical tool that could predict psychological scores from brain metabolic data. Further work could simulate surgical lesions within this model, and quantify its power to forecast the behavioural effects of any surgical intervention before it takes place.

## Supplementary Material

awaa032_Supplementary_Data

## Abbreviations

HADS =: Hospital Anxiety and Depression Scale
PPCA =: probabilistic principal component analysis
WAIS =: Wechsler Adult Intelligence Scale III
