# Supplementary material for: Metabolic lesion-deficit mapping of human cognition
Source: Brain. 2020 Mar 23;143(3):877–90. doi: 10.1093/brain/awaa032 (PMC7089650; doi:10.1093/brain/awaa032)
Supplement: awaa032_Supplementary_Data [file awaa032_supplementary_data.pdf]

Metabolic lesion–deficit mapping of human cognition

SUPPLEMENTARY MATERIAL

Cohort description

All patients underwent evaluation for epilepsy surgery at the National Hospital for Neurology and Neurosurgery, Queen Square, between June 2006 and February 2011. Demographic and clinical details related to their epilepsy are shown in table S1.

Age during assessment (years)	Median	32.0
	Range (5th – 95th percentile)	18.1-52.6
	Missing (%)	0 (0)
Handedness	Right-handed (%)	135 (84.9)
	Left-handed (%)	19 (7.5)
	Ambidextrous (%)	3 (1.9)
	Missing (%)	2 (1.3)
Age of onset of epilepsy (years)	Median	12.0
	95% Range	0.5-36.6
	Missing (%)	10 (6.3)
Aetiology of Epilepsy	Unknown (%)	148 (93.1)
	Prior central nervous system infection (%)	6 (3.8)
	Prior central nervous system trauma (%)	1 (0.6)
	Missing (%)	4 (2.5)
Seizure types	Focal aware (%)	50 (31.4)
	Focal unaware (%)	129 (81.1)
	Focal to bilateral tonic clonic (%)	97 (61.0)
	Other (%)	19 (11.9)
	Missing (%)	6 (3.8)
Presumed epileptogenic zone	Frontal (%)	59 (37.1)
	Temporal (%)	79 (49.7)
	Parietal (%)	11 (6.9)

	Occipital (%)	3 (1.9)
	Non-localised (%)	25 (15.7)
	Missing (%)	6 (3.8)
18F-FDG PET scan clinical report	Normal (%)	56 (35.2)
	Abnormal (%)	100 (62.9)
	Missing (%)	3 (1.9)
Last recorded surgical outcome	No surgery	101 (63.5)
	Vagal Nerve Stimulation (%)	17 (10.7)
	Temporal resection (%)	22 (13.8)
	Extra-temporal resection (%)	17 (10.7)
	All surgical resection (%)	39 (24.5)
	External Trigeminal Nerve Stimulation (%)	1 (0.7)
	Missing (%)	2 (1.3)
Pathology (out of 39 resection specimens)	Non-specific (%)	13 (33.3)
	Hippocampal sclerosis (%)	7 (17.9)
	Focal Cortical Dysplasia (%)	15 (38.5)
	Other (%)	2 (5.1)
	Missing (%)	2 (5.1)

Table S1: Demographic and clinical details. Only adults were included with a minimum age of 18 years old. The vast majority had epilepsy with unknown aetiology with focal onset seizures which is typical of a cohort being considered for epilepsy surgery. Interictal ^{18}F-FDG PET imaging was clinically reported to be within the normal range in 35.2% of cases. In order to guide potential resection surgery, the attending team of clinicians review all clinical details and investigation results in order to determine the presumed epileptogenic zone – the cortical region that is indispensable for the generation of seizures(Rosenow and Lüders, 2001). This can be localised with varying degrees of accuracy and is summarised here in terms of the brain lobes affected, noting that individuals may have epileptogenic zones involving two or more lobes of the brain. Where the epileptogenic zone could not be localised beyond the hemisphere involved, or where the localisation was thought to be multi-focal, this is reported as non-localised. Of the individuals assessed, 24.5% underwent resection surgery and the anatomical pathology of the cortical resection specimens is also presented. The remaining patients either underwent less invasive surgical procedures such as Vagal Nerve Stimulation or External Trigeminal Nerve Stimulation or

continued with medical care only (63.5%). Subject numbers are shown followed by the percentage of the sample in brackets.

Psychological and psychiatric assessments

Patients undergo comprehensive psychological and psychiatric evaluations as part of their pre-surgical assessments which are reported below in table S2.

Educational Level	Special schooling (%)	6 (3.8)
	Mainstream school up to age 16 years (%)	43 (27.0)
	Mainstream school up to age 18 years (%)	38 (23.9)
	University or other higher education (%)	36 (22.6)
	Missing (%)	36 (22.6)
Psychological assessment	Normal or very mild impairment (%)	39 (24.5)
	Moderate localisable impairment (%)	56 (35.2)
	Moderate non-localisable impairment (%)	44 (27.7)
	Learning disability (%)	9 (5.7)
	Missing (%)	11 (6.9)
Psychiatric assessment	No issues highlighted (%)	69 (43.4)
	Affective symptoms but no treatment currently required or suggested (%)	41 (25.8)
	Affective symptoms requiring treatment (%)	28 (17.6)
	Missing (%)	21 (13.2)

Table S2: Psychological and psychiatric evaluations. The level of education is bound to affect psychological test scores and is reported in broad categories above focusing on the need for special schooling rather than mainstream schooling and the highest level of educational attainment. The assessing psychologist provides a global report of the nature and degree of psychological impairment. These reports have been categorised above according to their severity and whether the impairment pattern was confidently suggestive of localisable syndrome (e.g. dominant temporal lobe). Additionally, psychiatric evaluations performed by a Consultant Psychiatrist were available in the majority of patients revealing that at least 17.6% of patients had active significant psychiatric disease where treatment was suggested or ongoing. This more sever cohort dominantly comprised of long-standing depression and/or anxiety and included patients with episodes of deliberate self-harm.

Psychological test descriptions

Wechsler Adult Intelligence Scale (WAIS) (Wechlser, 2008). Version III was used. This provides a verbal IQ score based on 7 tests: vocabulary (name visually presented objects or define words), similarities (specify the similarity between two words), information (general knowledge), comprehension (questions about social situations or concepts), arithmetic (timed oral arithmetic problems) and digit span (repeat a verbally presented list of numbers, in original or reversed order). Performance IQ is scored according to the results of 5 further tests: block design (timed arrangement of blocks in pre-specified order), matrix reasoning (identify missing picture within an array, from a given selection), picture completion (identify the missing component of a picture), digit symbol-coding and symbol search. We note that some subsets (information, comprehension, block design, picture completion, digit-symbol coding and symbol search) were not performed with sufficient frequency across this cohort to perform a group-level analysis.

Warrington Recognition Memory test (Warrington, 1984). During the Words subtest training phase, 50 stimulus words are presented at a rate of 1 every 3 seconds. The patient is asked to rate each word to be pleasant or unpleasant. During the test phase, word-pairs are presented and the patient is required to identify the previously seen word. The Faces subtest follows the same format except that greyscale faces are used as stimuli. Raw scores for the Faces or Words components can range from 0-50.

List and Design learning. Both tasks form part of the Adult Memory and Information Processing Battery (AMIPB) and its successor the BIRT Memory and Information Processing Battery (BIMPB)(Coughlan *et al.*, 2007). For the List learning task, patients were required to immediately recall a 15 item list of words five times ('immediate recall', learning trials, one point for each word recalled, maximum total score 75) and to recall them again following the presentation of a distractor set of 15 words ('delayed recall trials', maximum score of 15). The design learning test requires the participant to reproduce a design comprising nine lines on a 4 by 4 grid. There are five learning trials and a delayed recall trial leading to a maximum of 45 points for learning and 9 for recall.

Verbal fluency. Patients were asked to name as many animals (semantic) or words beginning with a pre-specified letter (phonemic) in 1 minute. The score is the number of words generated (Bird *et al.*, 2004).

Hospital Anxiety and Depression Scale(Zigmond and Snaith, 1983): a 14 item questionnaire asking respondents to rate how they feel about various aspects of their life in the past week. Separate scores for anxiety and depression can be calculated from the ratings. Scores range

from 0-21. A score of 8-10 supports borderline/ mild depression, whilst a score of 11 or higher is clinically abnormal.

Test group	Test component	Median score	Score Range: 5th percentile	Score Range: 95th percentile	Missing (out of n=159 cases)
Wechsler Adult Intelligence Scale III	Vocabulary	8.0	4.0	14.6	21
	Similarities	8.0	4.0	13.0	20
	Arithmetic	8.0	3.0	14.0	42
	Digit Span	8.0	4.0	14.0	17
	Matrix Reasoning	10.0	5.0	15.0	52
	Verbal IQ	90.0	66.6	115.0	37
	Performance IQ	92.0	69.8	122.8	61
Memory	Delayed List Recall	9.0	4.0	14.0	6
	Delayed Design Recall	8.0	2.0	9.0	42
	Immediate List Recall	47.5	25.6	61.7	3
	Immediate Design Recall	31.0	15.5	44.0	39
	Warrington Recognition Memory test for Words	47.0	36.0	50.0	58
	Warrington Recognition Memory test for Faces	41.0	31.0	48.0	87
Fluency	Phonemic	12.0	5.0	23.0	18
	Semantic	18.0	8.0	28.4	16
Affect (Hospital Anxiety and Depression Scale)	Depression	5.0	1.0	13.0	49
	Anxiety	8.0	3.0	15.0	49

Table S1 Psychological tests included within the study. The median and range (5th and 95th percentile within this cohort) of the scores of the selected psychological measures are presented for the 159 patients included in this analysis. Scores are grouped into 4 broad areas for ease of interpretation. Although neuropsychological assessment relies on a commonly used battery of tests, each patient undergoes a subset of these based on clinical need and judgement and so test coverage across patients naturally varies. These missing data were subsequently singly imputed using probabilistic principal component analysis (see methods). IQ: Intelligence quotient.

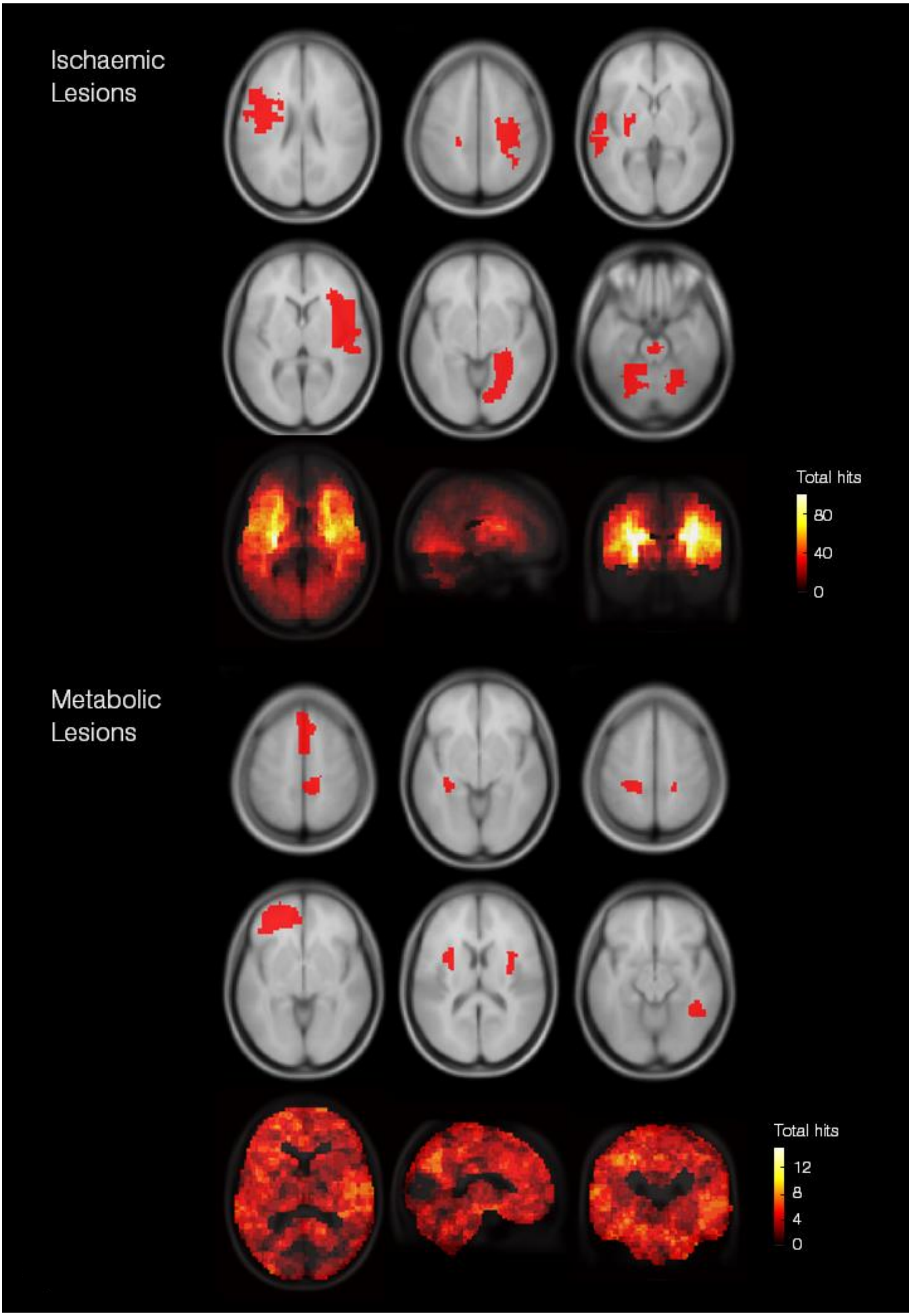
Ischaemic Lesions
Total hits
80
40
0
Metabolic Lesions
Total hits
12
8
4
0

Figure S1: Spatial distribution of ischaemic and metabolic lesions. Individual and summed ischemic and metabolic lesions are shown together to allow visual comparison. The top panel shows six exemplar ischaemic lesions derived from the 1333 automatically segmented, binary stroke lesion maps reported in (Xu *et al.*, 2017). Axial slices through the MNI-registered lesions are shown in red, overlaid on a template T1 MR image. The summed image of all lesions is shown immediately below presented as 3 orthogonal slices. For the purposes of visualisation and covariance testing only, metabolic ^{18}F-FDG PET lesions were defined to be regions of hypometabolism if they were below two standard deviations from the median values for each voxel. Six exemplar metabolic lesions are shown in the bottom panel. Axial slices through the MNI-registered binary lesion maps are shown in red, overlaid on a template T1 MR image. The summed image of all 159 lesions included in this study is shown immediately below presented as 3 orthogonal slices. Both Ischemic and metabolic lesions can be clearly distinguished as patches on individual images. Their distribution, however, is different. Ischemic lesions predominantly damage medial white matter structures and tend to spare the inferior temporal cortex bilaterally and the prefrontal cortex. Metabolic lesions are relatively uniformly distributed.

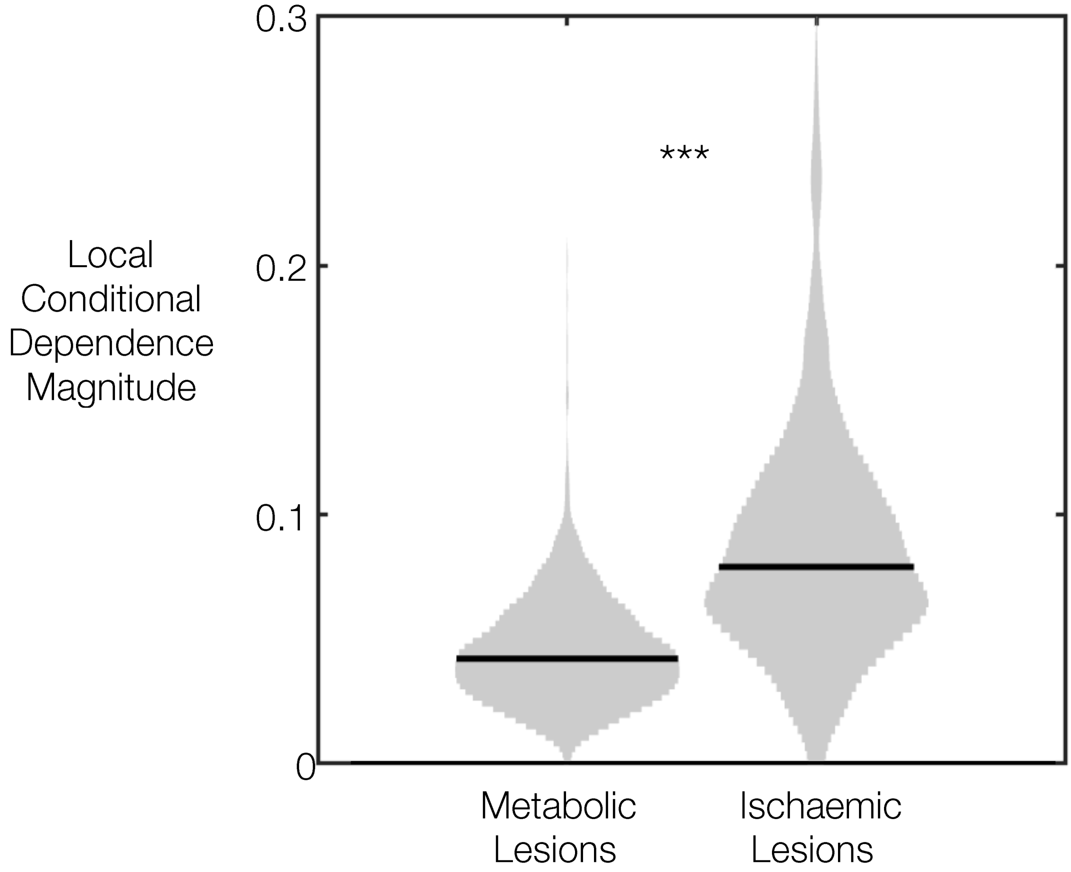

Figure S2: Local dependence of metabolic and ischaemic lesions. We generated two sets of voxel-wise spatial conditional probability vectors: one for a dataset of 1333 binary ischaemic lesions and one for a dataset of 159 binary metabolic lesions as described in the methods section. The magnitude of each vector corresponds to the conditional probability of a neighbouring voxel also being affected by a lesion and the direction corresponds to the relative location of the neighbouring voxel. The mean of these vectors - the normalised vector sum, also known as the mean resultant length - is a single *conditional dependency* vector that points towards the expected direction of greatest local dependence (and therefore potential inferential distortion). The magnitude of this vector is a probability and is, therefore, bounded between 0 and 1. Isotropic local dependencies will result in small randomly distributed mean vectors, whilst anisotropic dependencies will result in larger systematically directed vectors. The spatial distribution of voxel-wise conditional dependency vectors are shown in Figure 1). The distribution of the voxel-wise *magnitudes* for metabolic and ischaemic conditional dependency vectors is shown here in a violin plot, with the median value represented by a black line. A Kolmogorov–Smirnov test between the two distributions was significantly different (median metabolic = 0.042,

median ischaemic=0.079; ks statistic = 0.512, $p<0.0001$). The data confirm that metabolic lesions show lower systematic distortion of local covariance as compared to ischaemic lesions, and consequently are less likely to distort lesion-deficit inference.
